# Changes in Permanent Contraception Procedures Among Young Adults Following the *Dobbs* Decision

**DOI:** 10.1001/jamahealthforum.2024.0424

**Published:** 2024-04-12

**Authors:** Jacqueline E. Ellison, Brittany L. Brown-Podgorski, Jake R. Morgan

**Affiliations:** 1 Department of Health Policy and Management, University of Pittsburgh School of Public Health, Pittsburgh, Pennsylvania; 2 Center for Innovative Research on Gender Health Equity, Department of General Internal Medicine, University of Pittsburgh School of Medicine, Pittsburgh, Pennsylvania; 3 Department of Health Law, Policy, & Management, Boston University School of Public Health, Boston, Massachusetts

## Abstract

This cross-sectional study evaluates changes in tubal ligation and vasectomy procedures among younger adults following the *Dobbs v Jackson Women’s Health Organization* decision.

## Introduction

On June 24, 2022, the US Supreme Court’s decision in *Dobbs v Jackson Women’s Health Organization* overturned the constitutional right to abortion, permitting states to further restrict or ban abortion care. As of January 2024, 21 states have done so.^1^ This structural barrier to exercising control over pregnancy and childbearing will indirectly affect contraceptive decision-making.

Early research has documented increased demand for permanent contraception in the months following *Dobbs*, including tubal sterilization and vasectomy.^2,3^ This change may reflect fears of restricted access to abortion and/or contraception. However, no research, to our knowledge, has evaluated the differential effect of *Dobbs* on permanent contraception among men relative to women or among younger adults who are more likely to have an abortion and to experience sterilization regret.^4,5^ We therefore evaluated changes in tubal ligation and vasectomy following *Dobbs* among younger adults.

## Methods

We used data from the TriNetX platform for this cross-sectional study. These continuously updated medical record data are largely from academic medical centers and affiliated clinics in all 4 US census regions. We used an interrupted time series study design, fitting seasonally adjusted segmented autoregressive models to assess level and slope changes in procedure rates before (January 1, 2019, to May 31, 2022) and after (June 1, 2022, to September 30, 2023) *Dobbs*. Sensitivity analyses with a truncated pre-*Dobbs* observation window (April 1, 2021, to May 31, 2022) were conducted using Stata, version 17.1 (StataCorp LLC). This research was deemed exempt from review and the need for informed consent by the Boston University Institutional Review Board owing to the use of deidentifed patient data. We followed the (STROBE) reporting guideline.

Using monthly aggregate counts of tubal ligations and vasectomies, we calculated rates per 100 000 person-months among female and male patients aged 18 to 30 years. Individuals with an encounter for evaluation and management each month and no permanent contraception documented previously were included in the denominator. Visits for evaluation and management, tubal sterilization, and vasectomy procedures were identified using *Current Procedural Terminology* and *International Statistical Classification of Diseases, Tenth Revision* codes (eTable in Supplement 1). Two-sided *P* < .05 indicated statistical significance.

## Results

Observed permanent contraception procedure rates, estimates, and seasonally adjusted models for 22 063 348 person-months (36.9% male and 63.1% female) are presented in the Figure. Prior to *Dobbs*, the monthly permanent contraception rate increased by 2.84 and 1.03 procedures per 100 000 person-months among female and male patients, respectively (Table). *Dobbs* was associated with an immediate level increase of 58.02 procedures and 5.31 procedures per month among female patients. Among male patients, it was associated with a level increase of 26.99 procedures and no significant change in the number of procedures per month. Findings were robust to sensitivity analyses.

**Figure.**
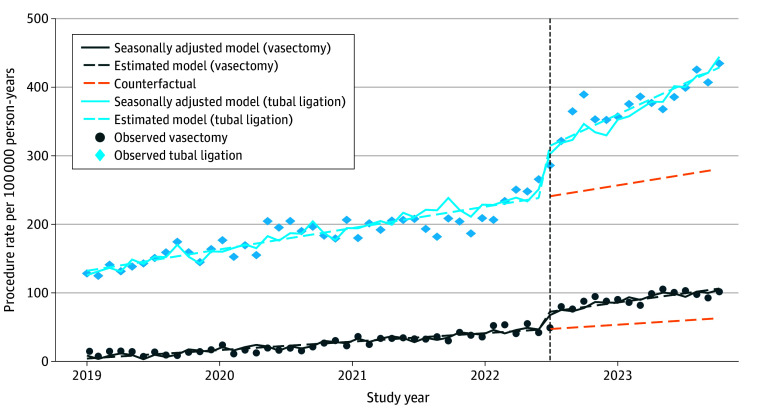
Monthly Time Series of Tubal Ligation and Vasectomy Procedure Rates Among Adults Aged 18 to 30 Years The dotted vertical line indicates the *Dobbs v Jackson Women’s Health Organization* decision.

**Table.** Changes in Permanent Contraception Procedure Rates Among Adults Aged 18-30 Years Following the *Dobbs v Jackson Women’s Health Organization* Ruling

Measure	Monthly procedures			
	Among female patients		Among male patients	
	Estimate (SE) [95% CI] per 100 000 person-months	*P* value	Estimate (SE) [95% CI] per 100 000 person-months	*P* value
Intercept	126.02 (8.13) [109.61 to 142.43]	<.001	8.57 (3.22) [2.03 to 15.06]	.01
Secular trend before *Dobbs*	2.84 (0.32) [2.19 to 3.50]	<.001	1.03 (0.09) [0.84 to 1.23]	<.001
Change in level after *Dobbs*	58.02 (23.41) [10.77 to 105.27]	.02	26.99 (7.27) [12.32 to 41.66]	.001
Change in trend after *Dobbs*	5.31 (2.15) [0.98 to 9.64]	.02	1.18 (0.7) [−0.22 to 2.59]	.10

## Discussion

We observed an abrupt increase in permanent contraception procedures among adults aged 18 to 30 years following *Dobbs*. The increase in procedures for female patients was double that for male patients. These patterns offer insights into the gendered dynamics of permanent contraceptive use and may reflect the disproportionate health, social, and economic consequences of compulsory pregnancy on women and people with the capacity to become pregnant.

This study has several limitations. The TriNetX platform does not capture state or health care organization identifiers. We were therefore unable to assess the potential outcomes of state abortion policy or account for changes in the sample attributable to fluctuations in the organizations contributing data over the study period. Additionally, our findings do not provide insight into the differential experiences of Black, Indigenous, Hispanic, disabled, immigrant, and low-income women, who disproportionately encounter interference and coercion in their contraceptive decision-making.^6^

The abrupt increase in permanent contraception rates may indicate a policy-induced change in contraceptive preferences. *Dobbs* may have also increased a sense of urgency among individuals who were interested in permanent contraception before the decision. Changes in contraceptive decision-making must be considered to understand the short- and long-term implications of *Dobbs* on reproductive autonomy.

## Notes

Supplement 1. **eTable.** Diagnosis and Billing Codes Used to Identify Tubal Ligation, Vasectomy, and Encounters for Evaluation and Management (E&M)

Supplement 2.
Data Sharing Statement
