# Error in Figure

**DOI:** 10.1001/jamahealthforum.2024.1164

**Published:** 2024-05-10

In the Research Letter titled “Changes in Permanent Contraception Procedures Among Young Adults Following the *Dobbs* Decision,”^1^ published April 12, 2024, the y-axis label in the Figure was incorrect; it should have read “Procedure rates per 100 000 person-months.” This article has been corrected.
