# Supplementary material for: Changes in Permanent Contraception Procedures Among Young Adults Following the Dobbs Decision
Source: JAMA Health Forum. 2024 Apr 12;5(4):e240424. doi: 10.1001/jamahealthforum.2024.0424 (PMC11065151; doi:10.1001/jamahealthforum.2024.0424)
Supplement: Supplement 1. — eTable. Diagnosis and Billing Codes Used to Identify Tubal Ligation, Vasectomy, and Encounters for Evaluation and Management (E&M) [file jamahealthforum-e240424-s001.pdf]

Supplementary Online Content

Ellison JE, Brown-Podgorski BL, Morgan JR. Changes in permanent contraception procedures among young adults following the *Dobbs* decision. *JAMA Health Forum*. Published online April 12, 2024. doi:10.1001/jamahealthforum.2024.0424

eTable. Diagnosis and Billing Codes Used to Identify Tubal Ligation, Vasectomy, and Encounters for Evaluation and Management (E&M)

This supplementary material has been provided by the authors to give readers additional information about their work.

© 2024 Ellison JE et al. *JAMA Health Forum*.

eTable. Diagnosis and Billing Codes Used to Identify Tubal Ligation, Vasectomy, and Encounters for Evaluation and Management (E&M)

	**Source**	**Codes**
Tubal ligation	ICD10CM	Z30.2
	CPT	0567T, 58565, 58600, 58605, 58611, 58615, 58670, 58671, A4264
	ICD10PCS	0U570ZZ, 0U573ZZ, 0U574ZZ, 0U577ZZ, 0U578ZZ, 0UL70CZ, 0UL70DZ, 0UL70ZZ, 0UL73CZ, 0UL73DZ, 0UL73ZZ, 0UL74CZ, 0UL74DZ, 0UL74ZZ, 0UL77DZ, 0UL77ZZ, 0UL78DZ, 0UL78ZZ, 0UT70ZZ, 0UT74ZZ, 0UT77ZZ, 0UT78ZZ, 0UT7FZZ
Vasectomy	CPT	55250
	ICD10CM	Z98.52[a]
E&M encounter	CPT	99202-99499

[a] Used only to exclude enrollees from the denominator

© 2024 Ellison JE et al. *JAMA Health Forum*.
