# Supplementary material for: Changes in Permanent Contraception Procedures Among Young Adults Following the Dobbs Decision
Source: JAMA Health Forum. 2024 Apr 12;5(4):e240424. doi: 10.1001/jamahealthforum.2024.0424 (PMC11065151; doi:10.1001/jamahealthforum.2024.0424)
Supplement: Supplement 2. — Data Sharing Statement [file jamahealthforum-e240424-s002.pdf]

Data Sharing Statement

Ellison. Changes in Permanent Contraception Procedures Among Young Adults Following the Dobbs Decision. *JAMA Health Forum*. Published April 12, 2024.
doi:10.1001/jamahealthforum.2024.0424

Data
Data available: No

Additional Information
Explanation for why data not available: The data that support the findings of this study come from the TriNetX research platform and sharing is not permitted by regulation.
